# Understanding xylose isomerase from *Burkholderia cenocepacia*: insights into structure and functionality for ethanol production

**DOI:** 10.1186/s13568-019-0795-4

**Published:** 2019-05-24

**Authors:** Igor P. V. Vieira, Gabrielle T. Cordeiro, Diego E. B. Gomes, Rafael D. Melani, Leonardo F. Vilela, Gilberto B. Domont, Rafael D. Mesquita, Elis C. A. Eleutherio, Bianca C. Neves

**Affiliations:** 1 Departamento de Bioquímica, Instituto de Química, Universidade Federal do Rio de Janeiro (UFRJ), Avenida Athos da Silveira Ramos, 149. Centro de Tecnologia, Bloco A, 5° andar, sala 537. Cidade Universitária, Rio de Janeiro, RJ CEP: 21.941-909 Brazil; 2 Institut de Science et d’Iengénierie Supramoléculaires, Université de Strasbourg, 8 Allée Gaspard Monge, BP 70028, 67083 Strasbourg Cedex, France; 3 Diretoria de Metrologia Aplicada às Ciências da Vida, Instituto Nacional de Metrologia, Qualidade e Tecnologia, Duque de Caxias, 25.250-020, Brazil; 4 CAPES Foundation, Ministry of Education of Brazil, Brasilia, 70.040-020, Brazil; 5 Present Address: Molecular Biology Laboratory, Biology Institute, Universidade de Brasília, Campus Darcy Ribeiro, Brasília, DF Zip code: 70910-900 Brazil

**Keywords:** Xylose isomerase, XylA, *Burkholderia cenocepacia*, Ethanol, Xylose fermentation, Enzyme kinetics

## Abstract

The inability of the yeast *Saccharomyces cerevisiae* to produce ethanol from xylose has hampered the biofuel production from lignocellulosic biomass. However, prior studies reveal that functional expression of xylose isomerase (XI) from *Burkholderia cenocepacia* (XylA_Bc_) in *S. cerevisiae* has remarkably improved xylose consumption and ethanol productivity. Yet, little is known about kinetic and structural properties of this enzyme. Hereby, a purified recombinant XylA was assayed in vitro, showing optimal enzyme activity at 37 °C and pH 7.2. The K_m_ of XylA for d-xylose was at least threefold lower than the K_m_ results for any XI published to date (e.g. XylA from *Piromyces* sp.). In addition, oligomerization behavior as a tetramer was observed for XylA in solution. Functional and structural comparative analyses amongst three microbial XIs were further performed as theoretical models, showing that xylose orientation at the active site was highly conserved among the XIs. Mg^2+^ ions anchor the sugar and guide its pyranoside oxygen towards a histidine residue present at the active site, allowing an acid–base reaction, linearizing xylose. Electrostatic surface analyses showed that small variations in the net charge distribution and dipole moment could directly affect the way the substrate interacts with the protein, thus altering its kinetic properties. Accordingly, in silico modeling suggested the tetramer may be the major functional form. These analyses and the resulting model promote a better understanding of this protein family and pave the way to further protein engineering and application of XylA in the ethanol industry.

**Electronic supplementary material:**

The online version of this article (10.1186/s13568-019-0795-4) contains supplementary material, which is available to authorized users.

## Introduction

The increasing concern about climate changes and its effects on human health has created a global effort for the development of new technologies based on renewable energy matrixes (Tibbetts 2015; Perera 2016; Ko and Lee 2018). In this scenario, ethanol is the preferred biofuel produced worldwide and has proven to have the lowest impact on current economies based on fossil fuels (Nielsen and Keasling 2016). Furthermore, the growing population creates an increasing demand for energy dedicated to transports, thus raising the pressure for fuel production expansion. However, the ethanol fermentation process based on sugarcane and starch as feedstock is already close to theoretical maximum efficiency (Carlos et al. 2011). Moreover, the expansion of croplands suffers social and political embargo. Then, the use of residual lignocellulosic biomass from sugarcane bagasse has gained great attention. After the pretreatment and hydrolysis steps, it is possible to retrieve large amounts of hexoses and pentoses from cellulose and hemicellulose polymers (Mosier et al. 2005). Xylose is the second most common sugar present on lignocellulosic hydrolysates. However, the inability of the yeast *Saccharomyces cerevisiae* to produce ethanol from xylose is one of the problems associated with the direct application of this technology (Stambuk et al. 2008).

Xylose metabolism in *S. cerevisiae* requires the uptake of the sugar by non-specific hexose transporters of the *HXT* family (Kruckeberg 1996). Then, xylose is reduced to xylitol by xylose reductase (XR; E.C. 1.1.1.307) and xylitol is further oxidized into xylulose by xylitol dehydrogenase (XDH; E.C. 1.1.1.B19). These reactions are NAD(P)H and NAD^+^-dependent, respectively. D-xylulose has to be phosphorylated into 5P-xylulose by xylulokinase (XK) in order to enter the pentose phosphate pathway (PPP). PPP mainly performs NADP^+^ reduction and the production of glyceraldehyde-3-phosphate (G3P) and fructose-6P that can be oxidized into pyruvate via glycolysis and further converted into ethanol.

The redox imbalance generated by XR/XDH pathway cannot be overcome due to the lack of transhydrogenase activity in *S. cerevisiae*, responsible for converting NADH to NAD(P)H, leading to cytosolic NADH accumulation (Bruinenberg et al. 1985). Attempts to clone fungi recombinant enzymes that use the same cofactors for conversion of d-xylose into d-xylulose have failed in producing an efficient strain capable of performing alcoholic fermentation of glucose and xylose simultaneously, without accumulation of by-products (xylitol and glycerol) (Matsushika et al. 2009; Gonc et al. 2014).

On the other hand, xylose Isomerase (XI; E.C. 5.3.1.5) pathway, used by some bacterial species to metabolize xylose (Hahn-Hägerdal et al. 2007), directly converts d-xylose into d-xylulose commonly using divalent metals as cofactors (Asboth and Naray-Szabo 2000; Hahn-Hägerdal et al. 2007). As reviewed in Moysés et al. (2016), the first functional recombinant XI expressed in *S. cerevisiae* was cloned from *Thermus thermophiles* and showed low activity at fermentation conditions. Another strain was successfully obtained by Kuyper et al. (2003), where the fungal XI from *Piromyces* sp. showed high activity but slow growth in xylose. Moreover, the same strategy using xylA from *Orpinomyces* was tested and likewise, high activities were observed but also had a slow growth in xylose (Madhavan et al. 2009). Nevertheless, XI from anaerobic bacterium *Clostridium phytofermentans* was expressed in an industrial *S. cerevisiae* strain with a similar K_m_ compared to *Piromyces* xylA and approximately three times smaller K_i_ for xylitol. However, the strain could not efficiently produce ethanol without xylitol accumulation.

Similarly, Vilela et al. (2015) were able to successfully express the recombinant XI from *Burkholderia cenocepacia* (XylA_Bc_) with high activity in *S. cerevisiae*. After adaptive engineering in xylose as a sole carbon source, the recombinant yeast presented one of the best fermentation results so far (Moysés et al. 2016). Under anaerobic conditions, glucose and xylose were simultaneously consumed while no xylitol was accumulated. Those results show the great potential of this strategy, considering that no further genetic modifications were required, as occurred in other constructions (Cai et al. 2012). For a better understanding of the model proposed for *S. cerevisiae* expressing XylA_Bc_, detailed analyses of enzymatic functionality and structure was still necessary.

In this work, the XylA_Bc_ was expressed in *Escherichia coli* and purified. In vitro assays with purified recombinant XylA_Bc_ exhibited a similar performance to that observed in vivo with yeast strains that were submitted to extensive metabolic and adaptive engineering (Cai et al. 2012). Functional analyses in different pH and temperature conditions were performed. Enzyme activity was measured in optimal conditions and kinetic parameters such as K_m_, V_max_ and K_cat_ were determined. The conditions used on in vitro assays were used to guide the creation of theoretical models by comparative modeling, in order to evaluate the overall structure, metal binding sites and xylose interaction at the putative active site. Molecular dynamics techniques were used to refine the models in conditions similar to those of enzymatic assays, aiming to determine any difference regarding the active site geometry, surface electrical charges, dipole moment and substrate interaction. The XIs from *Burkholderia cenocepacia*, *Piromyces sp* and *Clostridium phytofermentans* were analyzed, since those three microbial species present some of the best results published to date.

## Materials and methods

A synthetic DNA fragment containing the full-length *XylA* (GenBank accession no: AM747722) which encodes the enzyme Xylose Isomerase from *Burkholderia cenocepacia* J2315 strain (de Figueiredo et al. 2013) was obtained from Epoch Life Science (TX, USA). The gene was cloned into *Hin*dIII and *Bgl*II digested pTrcHis-B plasmid (Thermo Scientific, USA) and transformed in *E. coli* TOP10 [F^−^ *mcr*A Δ(*mrr*-*hsd*RMS-*mcr*BC) ϕ80*lac*Z ΔM15 Δ*lac*X74 *rec*A1 *ara*D139 Δ(*ara*-*leu*) 7697 *gal*U *gal*K *rps*L (Str^R^) *end*A1 *nup*Gλ] cells. Transformant cells were selected in LB broth with 200 μg/mL ampicillin. Protein expression was performed in 1-liter flasks containing 500 mL of LB broth and inoculated at 0.1 OD_600nm_ starting cell density. Cells were incubated at 37 °C under 160 rpm agitation until OD_600nm_ reached 0.6. Then, the culture was refrigerated to 18 °C and 500 μM IPTG was added to the medium for induction of protein expression for 16 h.

### Protein purification

IPTG-induced cells were washed with washing buffer (50 mM sodium phosphate, 300 mm sodium chloride, pH 7) and centrifuged at 2900×*g* for 30 min at 4 °C. Cell pellet was frozen at − 80 °C. In the day of use, thawed samples were disrupted with 10 sonication cycles of 1 min with 1 min intervals on ice-water bath using a 4 mm titanium micro-point (QR 300, Ultronique). The cell lysate suspension was centrifuged at 9100×*g* for 30 min at 4 °C and supernatant was collected. A Nickel (Ni^2+^) resin (Novagen^®^, Merck, USA) was equilibrated with 30 bed volumes of washing buffer. The cell lysate supernatant was incubated with the nickel resin for 30 min under gentle agitation on ice-cold water bath for His-tagged protein trapping. Then, the resin was added into a disposable Bio-Rad column and washed twice with 10 bed volumes of washing buffer. His-tagged proteins were eluted in a stepwise manner, with elution buffer (50 mM sodium phosphate, 300 mm sodium chloride, pH 7.5) supplemented with 100, 200 or 300 mM imidazole.

### Polyacrylamide gel electrophoresis (PAGE), protein concentration and determination

Protein expression and purification were initially analyzed in SDS-PAGE (5% stacking, 12% resolving gel). Sample loading dye contained 100 mM Tris–HCl pH 6.8, 8 M urea, 20% glycerol, 4% SDS, 0.2% bromophenol blue and 100 mM β-mercaptoethanol. Electrophoresis was performed at room temperature at a constant current of 0.06 A. Gel content was evaluated with Coomassie Brilliant Blue R-250 staining (BioRad). Eluted protein samples from the Ni^2+^ -resin were concentrated with Amicon Ultra 30K (Merck) by centrifuging at 4000×*g* at 4 °C for 10 min. The concentrated sample was fractionated into the Superdex 200 (GE Healthcare) resin with 1 mL/min flux of buffer (100 mM Tris–HCl, 20 mM NaCl, pH 7.5). The protein elution was followed by absorbance at 280 nm (ÄKTA Prime PLUS, GE Healthcare). Protein concentration after purification was assessed with BCA assay (Smith et al. 1985). The standard curve was made using BSA concentrations varying from 25 to 2000 μg/μL.

The molecular size and oligomerization of native His_6_-*XylA* was determined with a non-denaturing gradient PAGE (4–20% Mini-PROTEAN^®^ TGX™ Precast Protein Gel, BIO-RAD). Electrophoresis was performed as described above, with SDS-free sample and running buffers. A mixture of 10 recombinant proteins ranging from 10 a 250 kDa was used as standard (Precision Plus Protein™, BIO-RAD).

### Mass spectrometry

Protein bands were extracted from either native or SDS-PAGE and unstained on a solution of 30% methanol (v/v) and 10% acetic acid (v/v). Then, the sliced gel was incubated on a washing solution containing 50% acetonitrile (v/v) and 25 mM ammonium bicarbonate at pH 8.0 for 15 min. Protein samples were reduced on 8 M urea, 10 mM DTT and 25 mM ammonium bicarbonate solution at room temperature for 30 min. Then, the samples were incubated in a dark chamber in a 55 mM iodoacetamide and 25 mM ammonium bicarbonate solution for 30 min at room temperature. Trypsin (Promega) was added to the solution for proteolysis at 37 °C overnight. The digestion reaction was stopped with the addition of 1% formic acid (v/v). The peptides were cleaned using homemade C-18 stage tips (Rappsilber et al. 2007), dried, and resuspended in 0.1% formic acid (v/v) solution.

Peptides were fractionated using a nanoHPLC system Easy-nLC II (Proxeon) system on an in-house packed 2 cm × 150 μm i.d. pre-column (Reprosil-Pur C18-AQ, 5 μm, 120 Å, Dr. Maisch), and 15 cm × 75 μm i.d. analytical column (Reprosil-Pur C18-AQ, 3 μm, 120 Å, Dr. Maisch) coupled to an LTQ Velos Orbitrap (Thermo Fisher Scientific). Chromatography was performed at 300 nL/min. flow rate with 95% water, 5% Acetonitrile (ACN) and 0.1% formic acid (FA) as mobile phase A and 85% ACN, 15% water, and 0.1% FA as phase B. Two technical replicates of each sample were performed in optimized gradient of 55 min. Mass spectra were acquired by Tune and Xcalibur software operating in data-dependent acquisition (DDA) mode, switching between full scan MS1 (60,000 resolution at 200 m/z, 100 ms accumulation time, AGC 1 × 10^6^ ions, range 375–1800 m/z) and MS^2^ (15,000 resolution at 200 m/z, 200 ms accumulation time, AGC 10^5^ ions, range 100–2000 m/z). MS^2^ spectra were obtained by HCD fragmentation of the 10 most intense ions using 30 normalized collision energy.

Peptide spectrum match (PSM) search was performed using Comet search engine from PatternLab for Proteomics v4.0 (Carvalho et al. 2016) against forward and reverse protein sequences from *E. coli* and *B cenocepacia*, downloaded from UniProtKB on January 31, 2017, and containing 34,214 entries. Carboxamidomethylation of cysteines was set as a fixed modification and oxidation of methionine was set as a variable modification. Semi-tryptic peptides were considered with a maximum of 3 missed cleavages and mass tolerance for MS^1^ was set at 40 ppm. Resultant peptides were processed and evaluated by Search Engine Processor (SEPro) from using the following filter parameters: 10 ppm deviation from theoretical peptide precursor, peptides longer than six amino acid residues, minimum number of peptides per protein 3, Delta CN of 0.001, and a 1% estimated protein-level FDR.

### Enzymatic assays

XI activity was measured according to Brat et al. (2009). A solution containing 195 μg/mL of purified XI was incubated at the desired working temperature until the moment of use. Assays were performed in a quartz cuvette with 1 cm optical path. A reaction mixture containing 0.23 mM NADH, 10 mM MgCl_2_, 2 U/mL sorbitol dehydrogenase and 29.25 μg of XI in 100 mM Tris–HCl (pH 7.2) was fresh made for a final volume of 1 mL and incubated for 5 min at temperatures varying from 25 to 37 °C. The assay was performed in a quartz cuvette with 1 cm optical path. The reaction started with the addition of d-xylose and then followed for 30 min by measuring the oxidation of NADH at 340 nm until its complete oxidation to NAD^+^. Variations of d-xylose concentration from 10 to 500 mM were used to determine kinetic parameters. The protein theoretical isoelectric point (pI) was calculated using ProtParam Tool (http://web.expasy.org/protparam/) in order to determine the pH interval for activity measurement. From that, optimal pH was found by varying the buffer pH from 5.8 to 7.6. For pH values between 5.8 and 7.0, it was used a 100 mM Bis–Tris + 70 mM NaCl buffer. The ionic strength of each buffer was calculated for each condition to remain constant at 87 mM. Xylitol inhibition assay was done by measuring enzyme activity in the presence of 125 mM xylose and 10–50 mM xylitol at pH 7.2, 37 °C.

### Protein tertiary structure prediction

Templates were chosen over the Protein Databank (PDB) based on their % identities and coverage to sequences using the BLAST tool (NCBI). For homology modeling, a multiple sequence alignment between target and templates were generated using MUSCLE (Edgar 2004) and MODELLER (Martí-Renom et al. 2000; Webb and Sali 2014) salign script. Alignments were manually checked for overall quality and edited when necessary. Modeling of the tertiary structures was performed by MODELLER v9.17 with 500 models generated. In order to choose the best model, Modeller’s objective function, DOPE score and Z-score (normalized DOPE) were used to pick the best models from the pool. Each model quality was assessed with PROCHECK for overall structure geometry, ERRAT for non-bonded interactions statistics and VERIFY 3D for compatibility of the atomic model of tertiary structure with its primary structure (Bowie et al. 1991; Lüthy et al. 1992; Laskowski et al. 1993). Identified anomalies in loop regions were fixed by running MODELLER’s Loop refining protocol. RMSD was calculated with PyMol v1.8.4.

### Molecular dynamics and energy minimization

Structural models for *B. cenocepacia, C. phytofermentans* and *Piromyces* sp. were positioned to 1a0c, 1a0d, 1s5 m and 1s5n crystallographic structures. Cyclical d-xylose structure was obtained by modifying the glucose (removal of the methyl group and addition of H) from 1s5 m and transferring the coordinates to the constructed models. Water molecules from 1a0c crystal structure were preserved, except for HOH 494, 495, 496, 639 (chain A), 527, 528, 529, 671 (chain B), 525, 526, 527, 670 (chain C), 558, 559, 560, 702 (chain D) that were overlapping xylose atoms at the active site.

Amino acid side chain protonation state was determined according to PROPKA algorithm (Olsson et al. 2011) for pH 7.0. Structures were prepared with AMBER 14 program package (Simmerling 2015). Topology parameters for protein construction were obtained from AMBER 14SB force field. Xylose topology parameters were obtained from GLYCAM 06j-1 force field (Kirschner et al. 2008). The system was involved in an octahedral water box with 15 Å distance from sides and neutralized with Na^+^ or Cl^−^ when necessary.

The model structures were relaxed before molecular dynamics simulation with the following protocol: (i) structural minimization with position restrictions at 100 kcal/mol for heavy atoms present on the protein and xylose; (ii) structural minimization with position restrictions at 10 kcal/mol for protein backbone and xylose heavy atoms; (iii) whole system minimization with weak positioned restrictions at 1 kcal/mol for the complex heavy atoms. Then, the system temperature was increased from 0 to 100 K with the canonical ensemble (NVT) for 20 ps. Next, it was used the isothermal-isobaric (NPT) ensemble from 100 to 29,815 K for 20 ps. Both were controlled with Berendsen temperature protocol (Berendsen et al. 1984). After the system heating, the system went through 250 ps at 1 kcal/mol for restriction density equilibration. Finally, the system went through another cycle of 250 ps for heavy atoms restriction equilibration at 0.1 kcal/mol. All production cycles were performed by approximately 20 ns.

### Electrostatic surface and electric field vector $\left( {\mathop {\text{E}}\limits^{ \to } } \right)$

The file preparation for electric field calculations was done using the online software PDB2PQR (Dolinsky et al. 2004). The protein topology parameters were calculated using PARSE force field. Amino acid side chain protonation state was designated with PROPKA software at the following pH: 7.2 (*B. cenocepacia)* and 7.5 (*C. phytofermentans* and *Piromyces* sp.). Electrostatic field calculation and visualization were done on VMD v1.9.3 with APBS v1.4.1 plugin. Grid boxes were automatically calculated and kept at 298.15 K for *C. phytofermentans* and *Piromyces* sp. and 310.15 K for *B. cenocepacia*.

### Statistical analysis

All values obtained during enzymatic assays were analyzed on GraphPad Prism 6 and were considered statistically different on a 95% confidence interval using ANOVA test. All assays were performed as two independent replicates with three technical replicates. Results presented are the mean values of the assay replicates with standard error values.

## Results

### Purification and oligomerization analysis of XylA_Bc_, a XI from *Burkholderia cenocepacia*

**Fig. 1:**
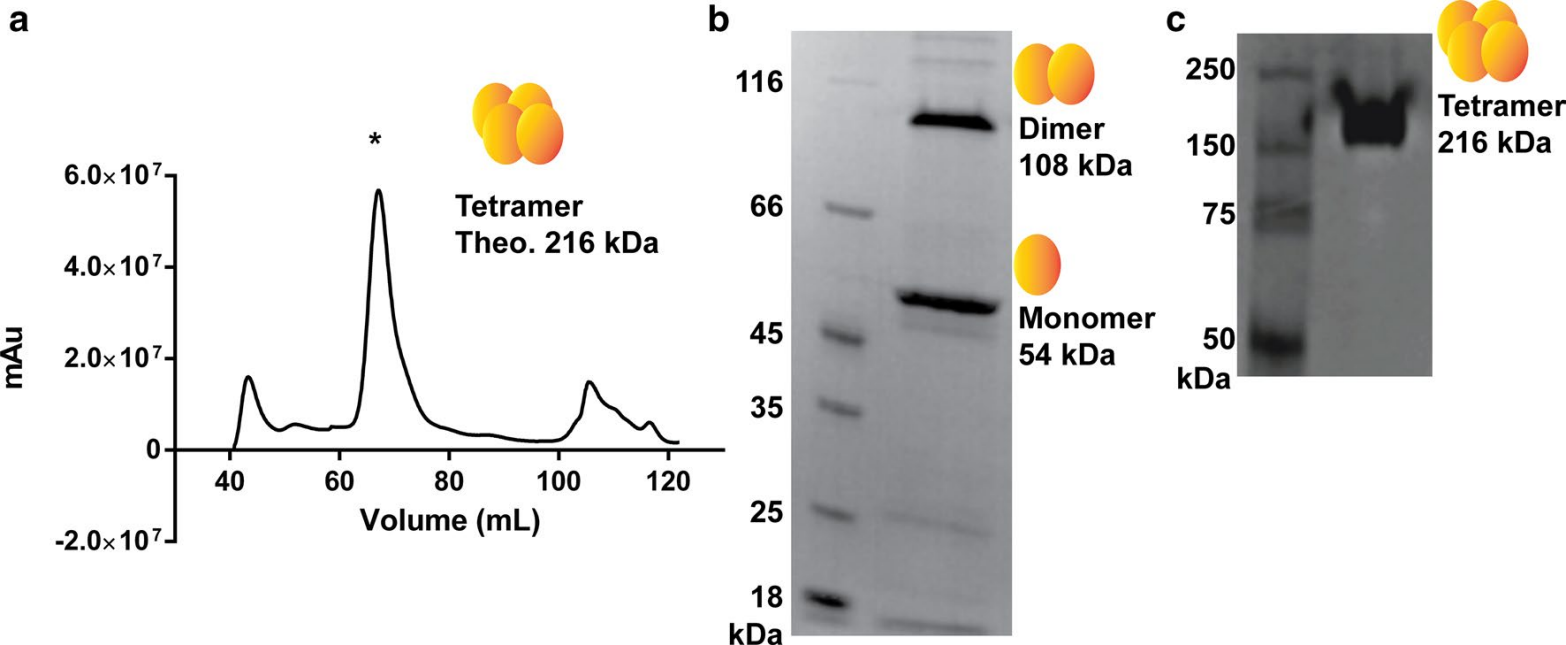
Purification and oligomerization behavior of a recombinant XylA from *B. cenocepacia*. **a** Size exclusion chromatogram obtained from Superdex 200 revealed a major peak. **b** Two major bands are observed on SDS-PAGE after size exclusion chromatography, revealing molecular sizes consistent with the monomer (ca. 54 kDa) and dimer (ca. 108 kDa) of His_6_XylA fusion. **c** Native 4–20% gradient PAGE gel shows a single protein band of ca. 216 kDa, when all His_6_XylA-containing fractions were pooled in a single sample. mAu, milli absorbance unit. *XylA containing fraction

To assess the functional properties of XylA_Bc_, the gene was cloned into pTrcHis plasmid under the control of P_Trc_ promoter, generating pTrcHis-XylA_Bc_ vector. *E. coli* TOP10 cells were transformed with the pTrcHis-XylA_Bc_ vector and the induction of protein expression was performed in Lysogen broth supplemented with 0.5 mM IPTG, at 18 °C for 16 h. The induction of XylA at a low temperature provided high yields of soluble protein and was capable of preventing the protein wrong folding and consequent precipitation. Expression of the His_6_-XylA fusion protein, with a theoretical mass of 53,598.67 Da, was analyzed on SDS-PAGE after cell lysis and sample enrichment on a Ni^+2^-resin (Additional file 1: Figure S1). The protein presented a band of the expected size (~ 54 kDa) after His-trap resin enrichment. The fractions containing His-tagged XylA were pooled and loaded onto a Superdex 200 resin column for size-exclusion chromatography. The His-tagged XylA_Bc_ eluted in a major peak, at 70 mL (Fig. 1a), as identified by digestion with trypsin. The resulting peptides were submitted to LC–MS/MS and eight proteins with maximum parsimony were identified. The best-scored protein (12,795.258 Xcorr) with 100% coverage was XylA_Bc_ (B4ENA5) and all the other 7 were identified with lowers scores, spectrum counts, and coverage (see additional files). Hence, XylA_Bc_-containing samples were analyzed on SDS-PAGE for approximate size estimative. Interestingly, even under denaturing conditions, XylA appeared as two band sizes of approximately 54 kDa and 108 kDa (Fig. 1b). Then, the most intense peak was loaded on a 4–20% gradient native-PAGE and ran as a single band of ca. 208 kDa, therefore compatible with a homotetramer (Fig. 1c). In order to confirm if the observed protein bands corresponded to the XyIA monomer, dimer, and tetramer, they were excised from the respective gels (Fig. 1b, c), digested with trypsin, and submitted to LC–MS/MS analysis. In all the three bands, the top score, coverage, spectrum count, and unique peptides pointed towards XI (B4ENA5) as the top1 identified protein, the same identified in the purified sample from His-trap enrichment step.

### Functional analysis of XylA_Bc_

**Fig. 2:**
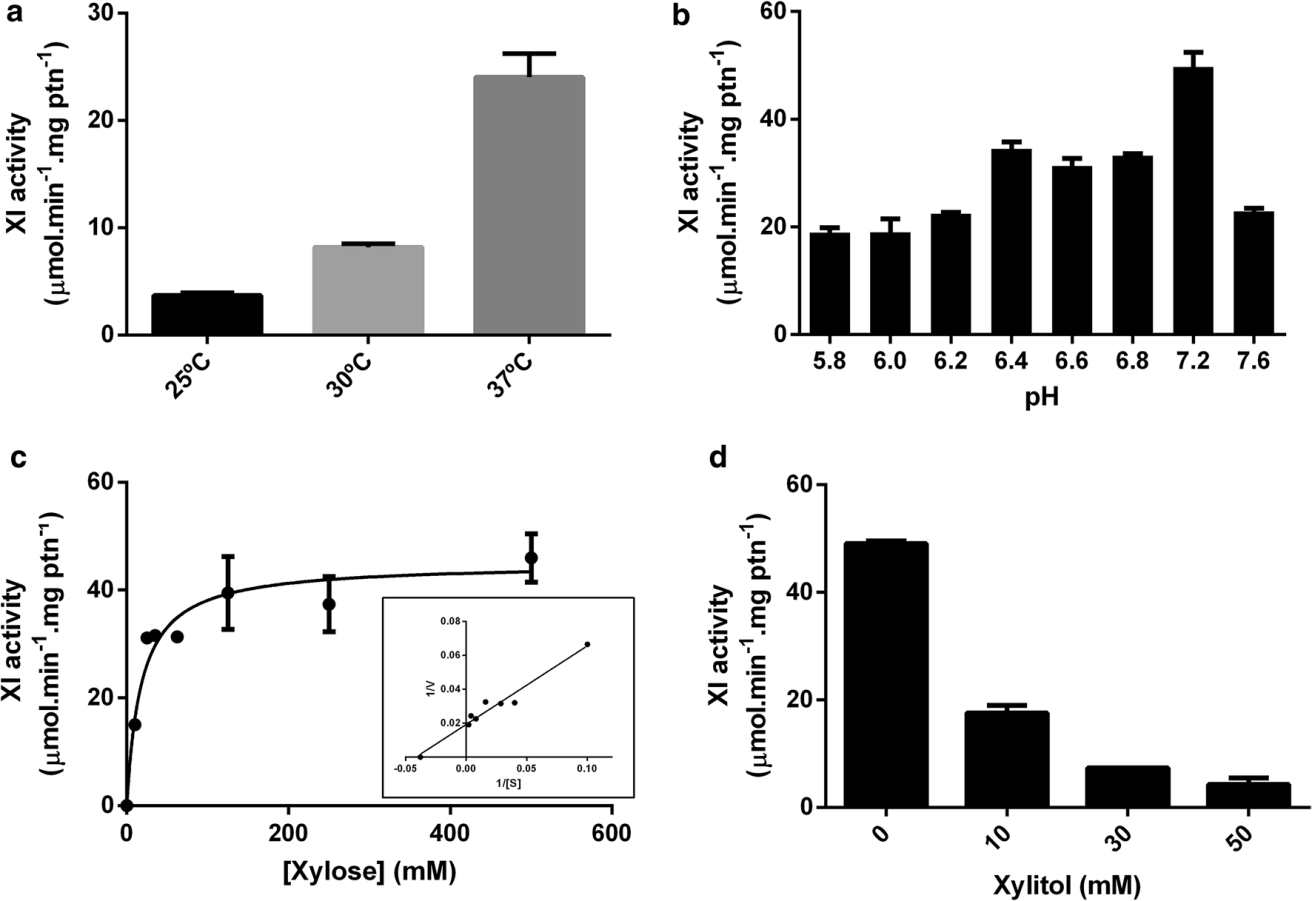
Functional analysis of XI from *B. cenocepacia*. **a** XI activity at different temperatures. **b** XI activity at different pH values. **c** Initial velocity dependence on the substrate concentration. **d** Xylitol inhibition with 125 mM d-xylose. Data shown are representative of the average of two independent replicates. Column bars represent the assay standard error of a technical duplicate

The XI specific enzymatic activity of the purified XylA_Bc_ samples was investigated. In order to find the optimal kinetics conditions, xylose isomerization was measured in a range of pH and temperatures. To find the best working temperature, buffers were kept at pH 7.5 and temperature varied from 25 to 37 °C. The temperatures used in the assays were chosen since they are generally the default temperatures used in sugarcane fermenters (Carlos et al. 2011). It was found that temperature variation has a great impact on enzymatic activity, as expected (Fig. 2a). The highest activity was 22.47 μmol min^−1^ mg ptn^−1^ at 37 °C. On each subsequent lower temperature, protein activity was lowered and reached 8.42 μmol min^−1^ mg ptn^−1^ at 30 °C and 3.53 μmol min^−1^ mg ptn^−1^ at 25 °C. Further assays used the optimum temperature (37 °C). To evaluate the influence of pH on XI activity, reaction mixes were buffered on a pH range from 5.8 to 7.6, varying − 1 pH below and +0.8 pH above the predicted isoelectric point (pI) and keeping all protein content soluble. Enzyme activity revealed to be substantially altered by pH variations as small as 0.2. The highest conversion rate was found at pH 7.2 being 51.61 μmol min^−1^ mg ptn^−1^ (Fig. 2b). Then, with the optimal conditions established, the K_m_, V_max_ and K_cat_ were calculated from the non-linear fit of a Michaelis–Menten equation. The apparent K_m_ for d-xylose is 17.08 ± 5.45 mM and V_max_ is 44.97 ± 2.06 μmol min^−1^ mg ptn^−1^ (Fig. 2c). The calculated K_cat_ for XylA_Bc_ was 19.02 ± 0.87 min^−1^. In addition, xylitol inhibition on this protein was evaluated since this by-product is one of the biggest concerns on using the XI strategy for ethanol production. The impact of the xylitol on XylA_Bc_ activity was tested on minimal xylose concentration of 7 times the K_m_ (125 mM), a guarantee that the V_o_–V_max_. Xylitol concentration varied from 10 to 50 mM. The smaller concentration of xylitol tested was capable of reducing the enzyme activity by 67% while 30 mM and 50 mM have reduced the enzymatic activity by 85% and 93%, respectively (Fig. 2d). The calculated IC_50_ for xylitol was 3.75 mM.

### In silico structural analysis of XylA

**Fig. 3:**
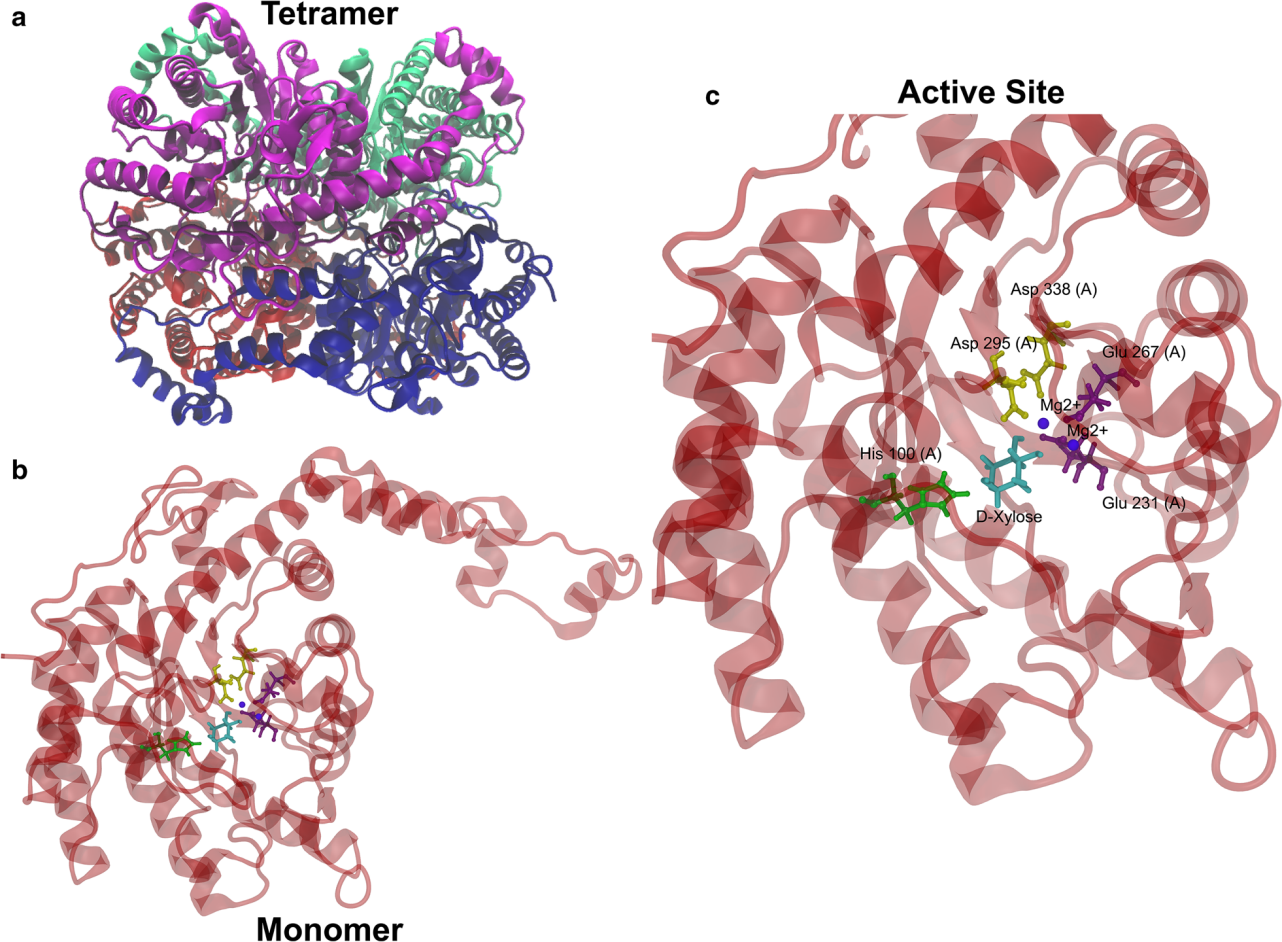
Xylose isomerase from *B. cenocepacia* predicted structure. **a** Overall structure of XylA_Bc_ as a tetramer. **b** Monomer structure of XylA_Bc_. **c** Active site representation

The structural model of XylA_Bc_ was investigated in order to determine whether environmental or structural factors could have a major impact on the protein activity in vivo (Fig. 3a). Furthermore, for comparison reasons, theoretical models for *C. phytofermentas* and *Piromyces* sp. XIs were also created (Additional file 1: Figures S2, S3). Then, the structures were assessed for overall folding quality. The Ramachandran plot analysis of the models presented a majority of atoms in allowed regions (approximate average of all models 94%) and less than 0.5% in disallowed regions (Additional file 1: Figures S4–S6). Further RMSD calculation between models and templates were performed and showed a distance < 0.6 Å to all comparisons. Likewise, all three models were very similar among themselves (RMSD < 0.5 Å) (Additional file 2: Table S1). These results show that the models have a high conservation of their tridimensional predicted structures despite they have small variations in the geometry of the active site. This somehow could be related to the differences observed on their performance in vivo. Then, molecular dynamics simulations were performed in order to allow the energy minimization of the protein-substrate complex structures.

### Molecular dynamics and energy minimization of XI protein-substrate complexes

In order to obtain models likely to happen in vivo, enzymatic assay conditions such as temperature and pH were used to calculate the protonation states of amino acid residues during the simulation. These variables can heavily contribute to the enzymatic activity since it can alter the interaction between the protein and the substrate. The PDB crystallographic structure *1s5* *m* was chosen as template to obtain metallic ions and substrate coordinates. The reason for the use of this template was the appropriate cyclic form of glucose (β-d-glucofuranose) presented at the active site. The glucose structure present at *1s5* *m* was edited into a xylose (β-d-xylopyranose) prior to coordinates transposing to the models. This form of xylose is believed to be preferred for isomerization reaction (Asboth and Naray-Szabo 2000). The protein-ion-substrate complexes were finally created by the transposition of the metallic ions and xylose coordinates from *1s5* *m*-*xylose* structure into the XI constructed models for each species. Then, simulations were performed in a water box with approximately 120 Å^3^ kept at 295.15 K and 1 atm for 20 ns. The results were analyzed in two steps: (i) overall and secondary structure stability, and general thermodynamic properties: (ii) active site geometry, substrate interaction and orientation.

All simulation models were visually inspected and no significant conformational transition was observed. The global RMSD and RMSF calculations were used as a metric for structural stability. The overall RMSD analysis revealed a similar relaxation for all structures of approximately 2 Å from its initial position (Additional file 1: Figure S7). The overall protein RMSF analysis showed that the larger fluctuations are concentrated at the amino acid residues closer to the surface that are directly exposed to the solvent (Additional file 1: Figure S8). The substrate RMSF result shows the fluctuation of each atom’s position along the structure (Additional file 1: Figure S10). The wide range of the torsion angles allowed by the primary and secondary C–OH ligations can explain this phenomenon. It is noteworthy that there is little movement of the atoms on the pyranoside ring of the sugar. However, the hydroxyl radicals at positions 0, 15, 17 and 19 considerably vary their rotation during simulations. In addition, the simulation indicates that the protonated nitrogen from the imidazole HIS residue side chains is likely to act as a catalyst by donating a proton to the oxygen in the xylose pyranoside ring (Fig. 3c).

Since all models from *B. cenocepacia*, *C. phytofermentans* and *Piromyces* sp. had similar tridimensional structures even after relaxation, it was thought that small variations in the active site could explain the results obtained in vitro. Then, the average volume of the pocket that contains the xylose binding site was calculated. The results have shown that there is no significant difference between the pocket volume of each chain in a same protein or among each predicted XI model (Additional file 1: Figure S11). Therefore, this suggests that the pocket size do not limit the active site availability for substrate binding and that cannot explain the results obtained in the functional analysis.

Yet no significant difference has been observed in the active site geometry of the theoretical models created, XIs from each species can vary its primary sequence to some degree and therefore, its specific activity. Therefore, a thorough investigation on the physical and chemical properties of these proteins was still necessary.

### Electrostatic surface and dipole moment analysis

**Fig. 4:**
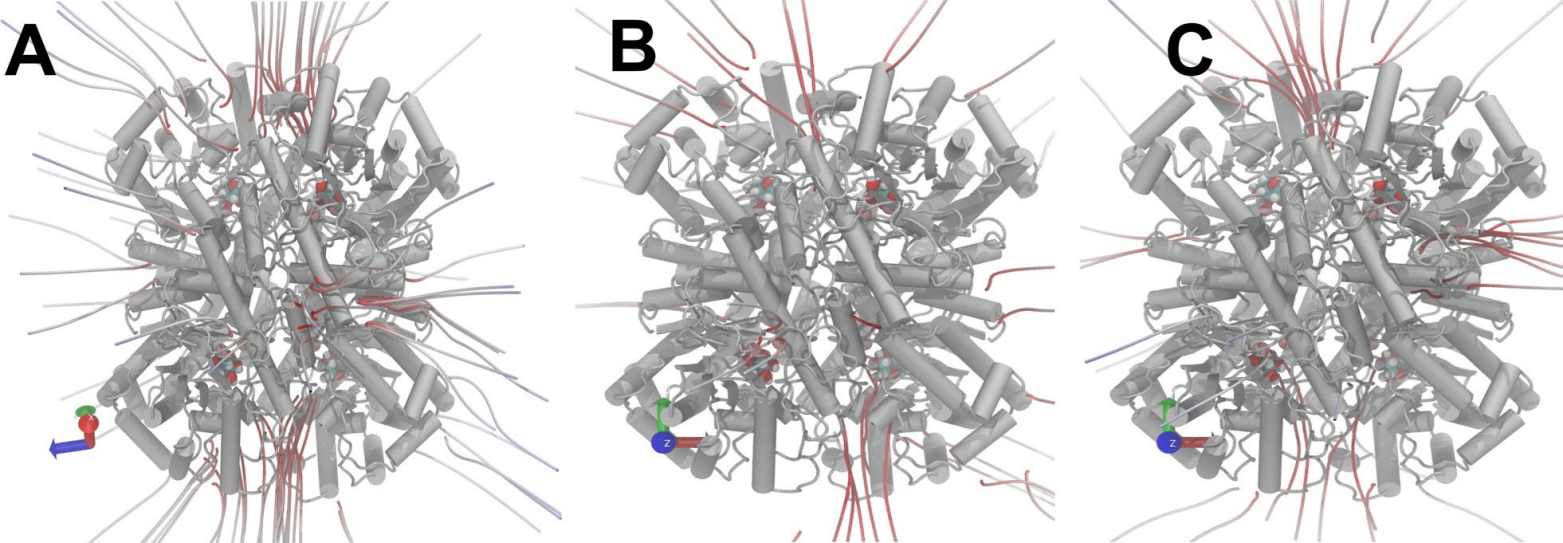
Electric field representation. **A** *B. cenocepacia*. **B** *C. phytofermentans*. **C** *Piromyces* sp. Red lines represent a negative charges. Blue lines represent positive charges. Grey lines represent neutral charges. Black arrows show the active site domains

**Fig. 5:**
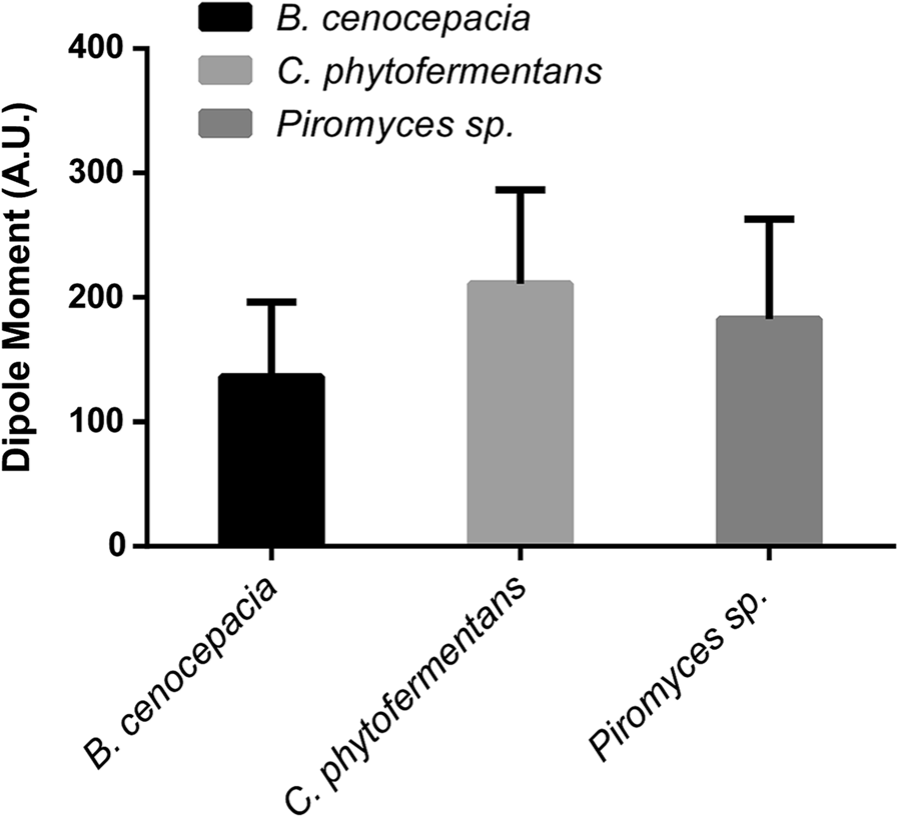
Dipole moment representing the net protein charge. All proteins atoms charge and topology were used to calculate the protein electric vector under similar conditions to the enzymatic assay. Statistical analyses of the data shown were performed with one-way ANOVA test and have shown a *p* value < 0.001. n = 11,407 models analyzed

Although the tridimensional structures are very similar, the sequence alignment only shows an identity about 50% (Additional file 1: Figure S12). Therefore, it is expected that residues substitutions could substantially alter the enzyme activity by altering electrostatic net charges along the protein tridimensional structure. Thus, the electrostatic surface of each protein was predicted for the models obtained after the molecular dynamics simulation. The three proteins analyzed showed a negative net charge distributed along the structure’s surface (Fig. 4). However, the charges can have different intensities for one protein, which directly correlates with the dipole moment of each protein. Then, variations in the electrical field vector $(_{ E }^{ \to })$ intensity and direction were determined with all models after the RMSF stabilization in the molecular dynamics. It was revealed that XylA_Bc_ has the smallest value between the three species analyzed, followed by *Piromyces* sp. and *C. phytofermentans*, respectively (Fig. 5). This result corroborates the data in Table 1, where the XI from *C. phytofermentans* has the highest K_m_ for xylose and the highest dipole moment intensity.

**Table 1:** K_m_ values of xylose isomerase from three different microbial species

Species	Km (mM)^a^	References
*B. cenocepacia*	17.08 ± 5.45	This work
*C. phytofermentans*	61.85 ± 3.41	Brat et al. (2009)
*Piromyces* sp.	49.85 ± 2.82	Kuyper et al. (2003)

^a^K_m_ values were obtained for d-xylose and are expressed with standard error calculated from a duplicate of independent assays

## Discussion

The purified XylA_Bc_ samples have shown multiple band sizes on SDS-Page denaturing conditions, which was a strong indication that the protein could have different oligomerization states, including a monomer and an SDS-resistant dimer. However, under native conditions, the protein has shown a single band of approximately 208 kDa. Taken together, these results strongly suggest that XylA_Bc_ is able to form homodimers and homotetramers, corroborating with previous structural studies of other bacterial XI (Peng et al. 2015). Considering that the protein sample of ca. 208 kDa appears as the only higher-order structure of XylA and was highly active in the XI enzymatic assays, we suggest that XylA may be able to assemble itself as homotetramer in vivo. Furthermore, the protein concentration used in the functional assays is consistent with protein concentration commonly found in yeasts (Milo 2013), showing that it is likely that XylA_Bc_ would present itself mostly as a homotetramer and that the homotetramer self-assembly observed in vitro is not a concentration dependent effect. The strong reduction power required to denature this protein shows its potential for industrial fermentation, where temperature variations can go from 30 to 42 °C depending on environmental conditions (Basso et al. 2008; Carlos et al. 2011).

To this date, most attempts to measure xylose isomerase (XI) activity have employed the use of crude cell extracts, through a coupled reaction with sorbitol dehydrogenase (SDH) to measure XI activity by monitoring and quantifying the oxidation of NADH (Kersters-Hilderson et al. 1987). Notwithstanding, it is difficult to accurately estimate the functional characteristics of the protein within the conditions of a complex biological extract. In the most used assay, the target protein is diluted in the cytoplasmic content within other NADH and NAD^+^ -dependent proteins that can affect the results of XI activity measurements. Therefore, it has been of great interest to evaluate the dynamics of this protein on both in vivo and in vitro conditions in order to determine the enzymatic activity, functionality and productivity. The XylA from *B. cenocepacia* was already proven to be functional in fermentation conditions and has shown to be one of the most promising XI performances described so far (Vilela et al. 2015). The functional analysis of this enzyme is necessary to understand the reasons why the sole expression of this protein was able to achieve such results, when other researchers had to perform multiple modification in strains to observe similar results (Cai et al. 2012). The XylA from *B. cenocepacia* showed a threefold lower K_m_ for xylose than the XI from *C. phytofermentans* and *Piromyces sp* xylA, one of the best microbial XI results published to date (Kuyper et al. 2003; Brat et al. 2009; Moysés et al. 2016). Those are remarkable results considering that a lower K_m_ can have a striking effect on the yeast xylose metabolism during fermentation. As XylA requires a smaller concentration of sugar to reach ½ V_max_, it is expected that it can compete for xylose with the yeast’s endogenous XR. It has been reported that *S. cerevisiae* endogenous XR has an approximate K_m_ for xylose of 27.9 mM (Jeong et al. 2001). Considering the results obtained in this work, it is possible to assert that the recombinant XylA_Bc_ is capable of diverting the metabolic flow towards XI pathway instead of the two-steps XR/XDH pathway. Therefore, it corroborates previous observation by our group (Vilela et al. 2015) showing that XylA_Bc_-engineered *S. cerevisiae* does not significantly accumulate by-products such as xylitol and glycerol, improving ethanol production.

Many researchers have found that xylitol is a potent inhibitor of XI, thus it is one of the biggest concerns of researchers using the XI strategy (Walfridsson et al. 1996; Klimacek et al. 2010; Zhang et al. 2012). On the other hand, Ha et al. (2011) brought up to light that xylitol inhibition may not be as severe in vivo as it is in vitro. Furthermore, Vilela et al. (2015) demonstrated that accumulation of xylitol is negligible during co-fermentation of glucose and xylose, thus it should not be a major concern for our model.

Further analyses revealed that the XI from *B. cenocepacia* K_cat_ for d-xylose is one of the highest ever reported and the specificity constant K_cat_/K_m_ is approximately 1056 s^−1^ M^−1^. It has been recently reported that mutations in the Ca^+2^/Mn^+2^ ATPase gene (*PMR1*) can alter the XI activity in vivo by increasing the overall concentration of divalent ions in the cell (Verhoeven et al. 2017). In this case, XylA from *Piromyces* sp. E2 was able to reach a K_m_ for d-xylose as low as 3.9 ± 0.2 mM and a K_cat_ of 7.8 ± 0.1 s^−1^, while the calculated K_cat_/K_m_ for XylA was 2000 s^−1^ M^−1^. Thus, the XylA from *B. cenocepacia* seems to have a lower specificity for the same substrate in comparison with XylA from *Piromyces* sp. However, the K_cat_ values indicate that XylA catalysis is more efficient in native conditions. It is possible that further modifications in our evolved strain could improve the XI activity even further as observed by Verhoeven et al. (2017).

Although recombinant XIs enzymatic activity greatly differ upon expression in *S. cerevisiae*, it was observed that all three XIs studied have similar tridimensional structures. It is commonly accepted that the primary sequence of a protein is responsible for the structure folding upon exposure to water in the cytoplasm. Therefore, it is expected that proteins with a large difference on their amino acid sequences should have different tridimensional structures as well. The XIs of *B. cenocepacia*, *C. phytofermentans* and *Piromyces* sp. showed amino acid identities of approximately 50% when compared to PDB 1s5 m sequence and the same can be observed in the multiple alignment of the three XIs (Additional file 1: Figure S12). The generated 3D models revealed a striking similarity among them with RMSD values smaller than 0.5 Å (Additional file 2: Table S1), despite the low average-value sequence identity. Molecular dynamics was used to minimize the energy and relax the protein-ion-substrate complex, in order to observe if small differences in the active site domain could explain the results observed.

The molecular dynamics simulations were able to show that regions exposed to the solvent have a greater degree of flexibility while regions closer to the active site undergo few structural modifications (Additional file 1: Figure S8). Furthermore, the xylose binding site residues are highly conserved among the analyzed species, as well as their tridimensional geometry. In either case, it is clear that the Mg^2+^ ion and the water molecules present at the active site have an important role on the substrate orientation within the active site domain. The presence of the ion and water molecules creates an anchoring platform caused by the anion–cation–anion charge distribution, similar to those observed in lysozymes and serine proteases (Asboth and Naray-Szabo 2000). Moreover, the oxygen present at the pyranoside ring of xylose is always aligned towards the HIS residue sidechains. The alignment of the pyranosidic oxygen creates a higher resonant movement in the molecule, reducing the energy barrier required for linearization of the substrate. Adding to this, the simulations have shown that the imidazolic hydroxyl group donates a proton for the oxygen and allows the linearization of the sugar. This kind of interaction has been previously described by Fenn et al. (2004) and Toteva et al. (2011) but with no clarity whether the HIS residue participated in the catalysis. We believe that our results corroborate with previous founds reported in the literature.

As no significant difference in the overall protein tridimensional structure was observed and all modeled proteins present the same type of interaction with the substrate, we decided to assess the electrostatic surface net charge and the protein dipole moment. Since the only reasonable difference between XIs was their primary amino acid sequence, any residues substitutions could alter the net charge of the protein electric field. The protein electrical surface directly influences the accessibility of the substrate to the active site. The results presented herein show a clear correlation between the dipole moment resultant from the electric field vector and the K_m_ shown by each XI. As the electric field gets more negatively intense, the K_m_ values are also increased. However, there is no canonical link stablished between the dipole moment of a protein structure related to its function. It is known that in some cases the electrostatic properties of a protein family are highly specific and can help directing the substrate towards the active site (Felder et al. 2007). We hypothesize that this may be one of the mechanisms behind the results observed in the enzymatic assays, since the compared structures are very similar. Further studies on phylogeny, amino acid conservation, structure and net charges are necessary in order to fully explain the correlation between these characteristics and the enzyme activity for this protein family.

## Additional files

**Additional file 1.** Additional figures.

**Additional file 2.** Additional tables.

## Availability of data and materials

Model PDB structures, mass spectrometry datasheet and dynamics simulation raw data are available and free of charge.
