# Supplementary material for: Understanding xylose isomerase from Burkholderia cenocepacia: insights into structure and functionality for ethanol production
Source: AMB Express. 2019 May 24;9:73. doi: 10.1186/s13568-019-0795-4 (PMC6534634; doi:10.1186/s13568-019-0795-4)
Supplement: Supplementary file 1 — Additional file 1. Additional figures. [file 13568_2019_795_MOESM1_ESM.pdf]

AMB Express

Understanding Xylose Isomerase from *Burkholderia cenocepacia*: insights into structure and functionality for ethanol production

Igor P.V. Vieira[a,1], Gabrielle T. Cordeiro[a], Diego E.B. Gomes[b,c,d], Rafael D. Melani[a], Leonardo F. Vilela[a]**,** Gilberto B. Domont[a], Rafael D. Mesquita[a], Elis C.A. Eleutherio[a], Bianca C. Neves[a]*

[a]Universidade Federal do Rio de Janeiro (UFRJ), Departamento de Bioquímica, Rio de Janeiro, 21.941-909, Brazil.

[b]Institut de Science et d'Iengénierie Supramoléculaires, Université de Strasbourg. 8 allée Gaspard Monge, BP 70028, 67083 Strasbourg Cedex, France.

[c]Diretoria de Metrologia Aplicada às Ciências da Vida - Instituto Nacional de Metrologia, Qualidade e Tecnologia. Duque de Caxias, 25.250-020, Brazil.

[d]CAPES Foundation, Ministry of Education of Brazil, Brasilia, 70.040-020, Brazil.

*Corresponding author:

Bianca Cruz Neves

Instituto de Química – Universidade Federal do Rio de Janeiro (UFRJ)

Avenida Athos da Silveira Ramos, 149. Centro de Tecnologia, Bloco A, 5° andar, sala 537. CEP: 21.941-909. Cidade Universitária, Rio de Janeiro, RJ, Brazil.

Tel.: +55 21 3938-7355

Fax.: +55 21 3938-7266

Email address: bcneves@iq.ufrj.br

---

[1]**Igor Patrick Vasconcelos Vieira**

Present address: Molecular Biology Laboratory, Biology Institute, Campus Darcy Ribeiro, Universidade de Brasília, Brasília, DF, Brazil. Zip code: 70910-900.

Additional Figures

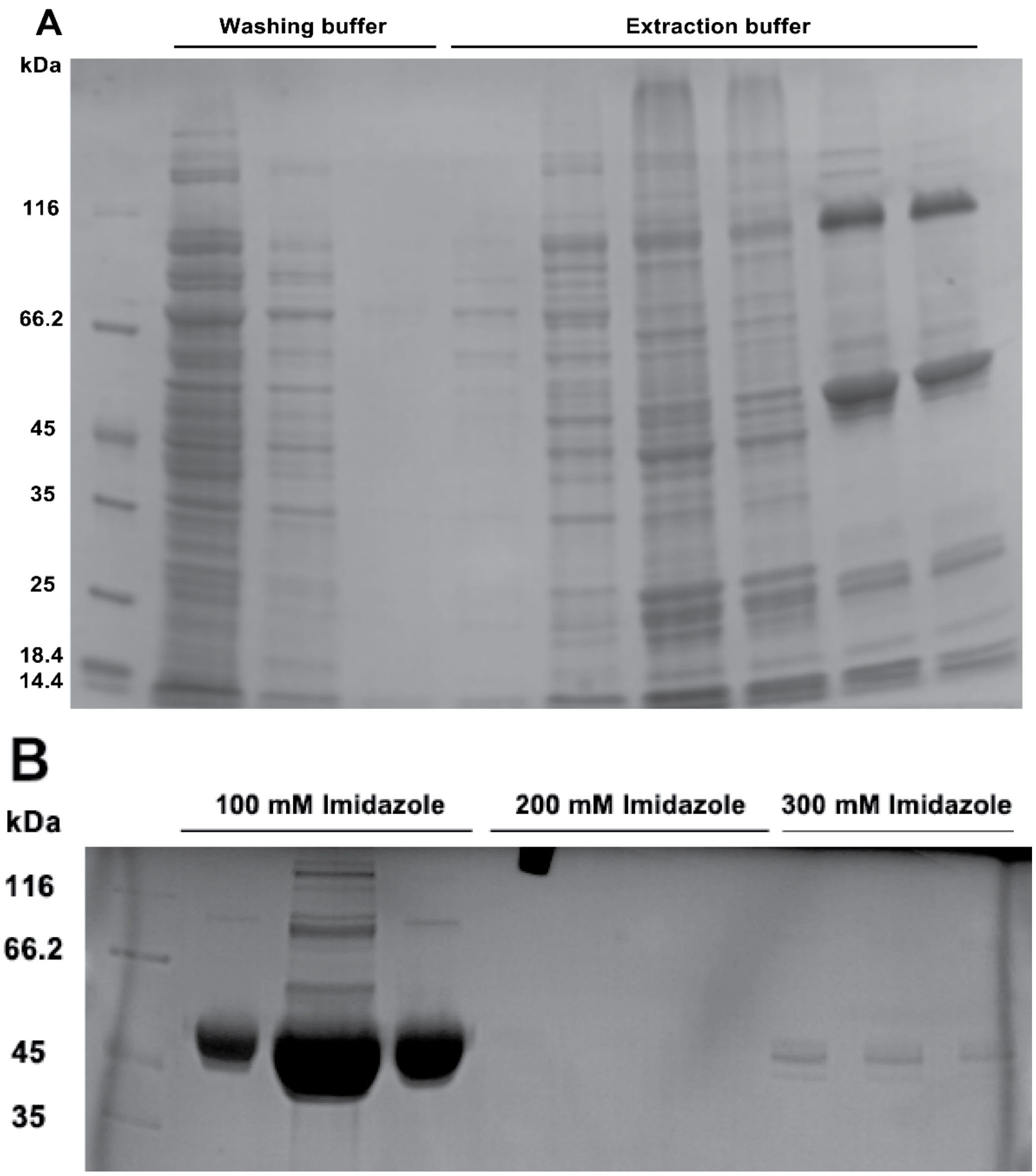

Figure S1. Protein extraction from *B. cenocepacia.* Only soluble proteins loaded onto the gel.

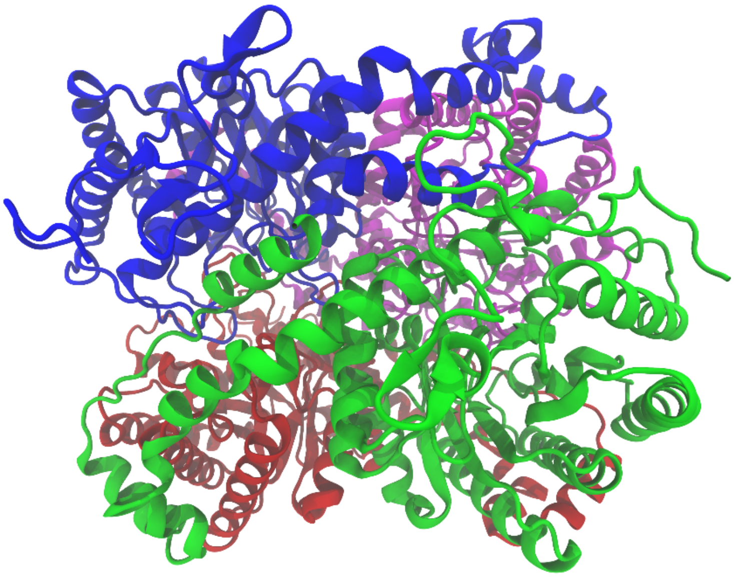

Figure S2. XylA predicted structure for *C. phytofermentans*.

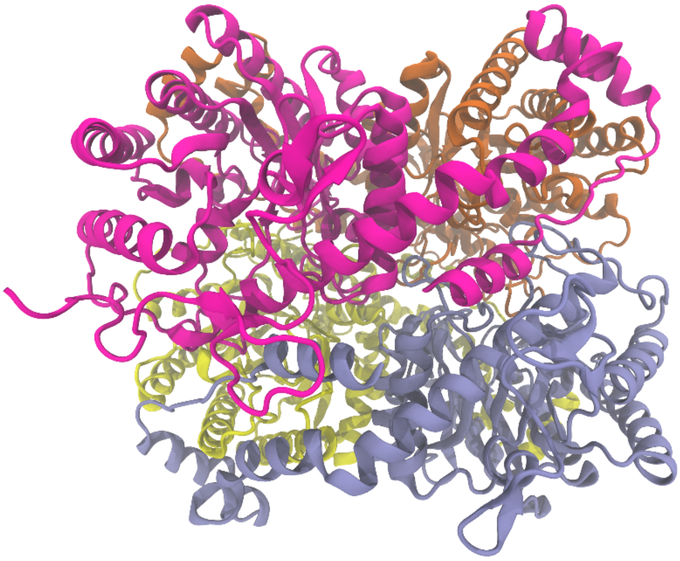

Figure S3. XylA predicted structure for *Piromyces sp.* E2.

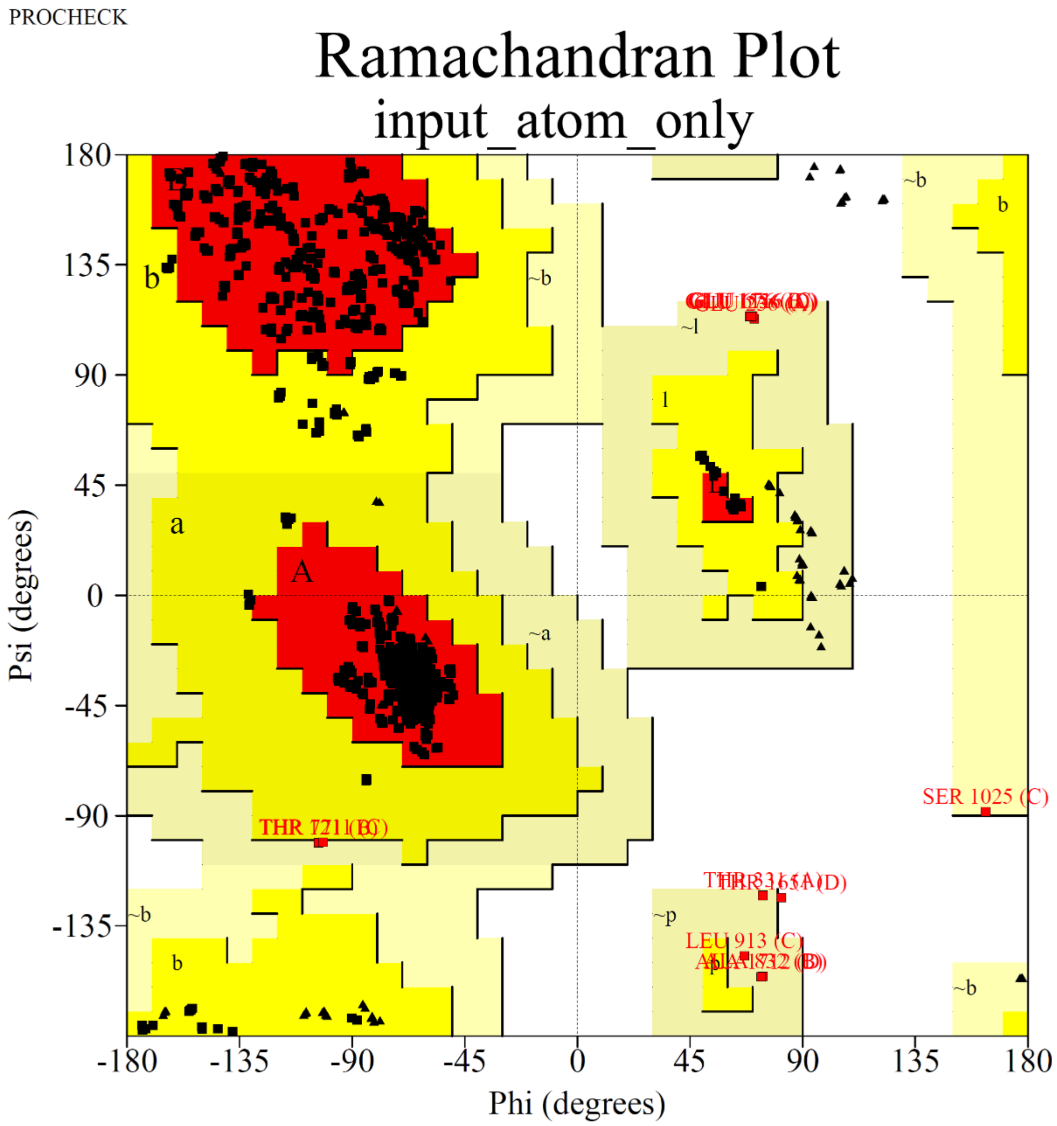

Figure S4. Ramachandran plot of *B. cenocepacia* XI theoretical model.

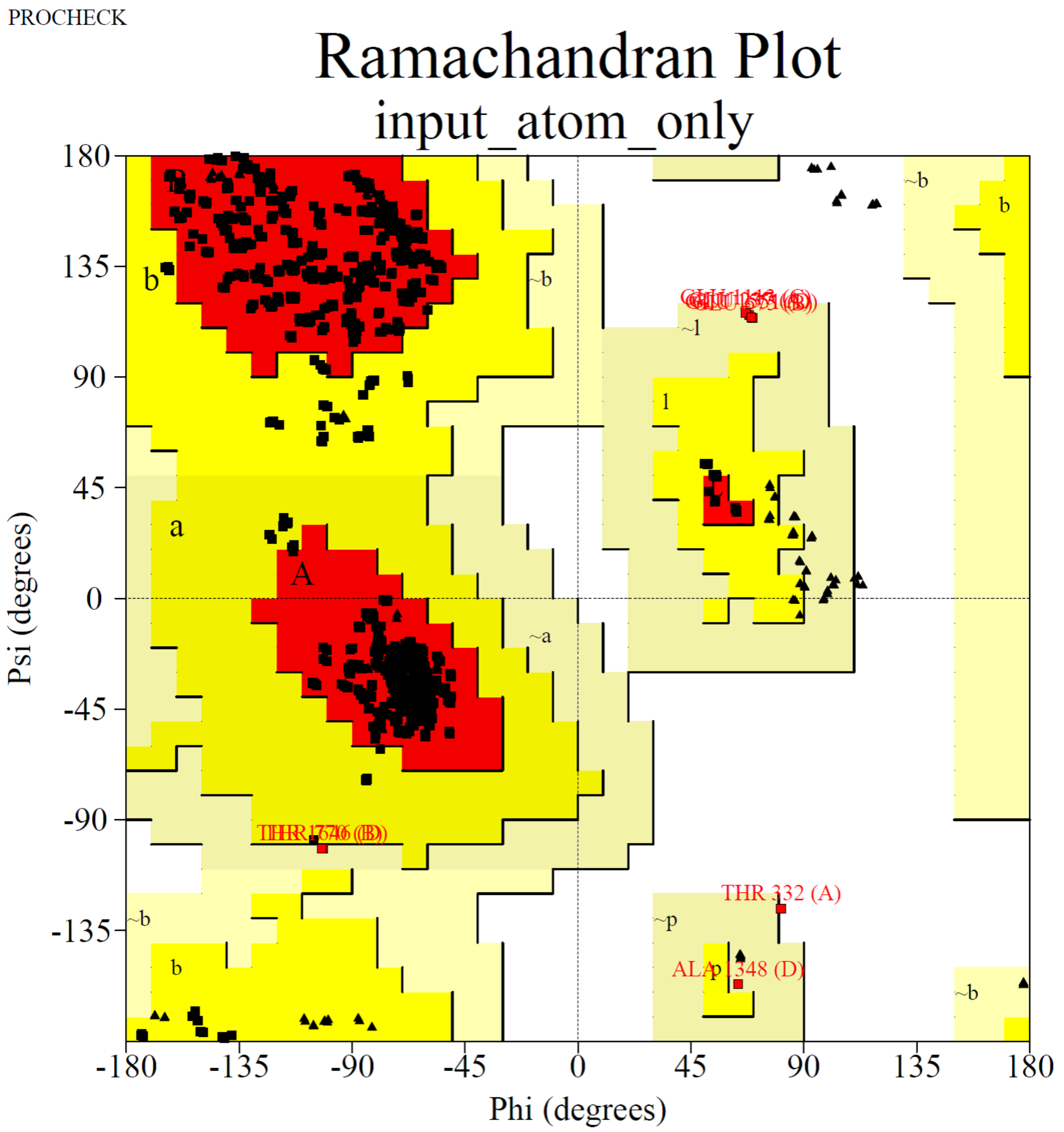

Figure S5. Ramachandran plot of *C. phytofermentans* XI theoretical model.

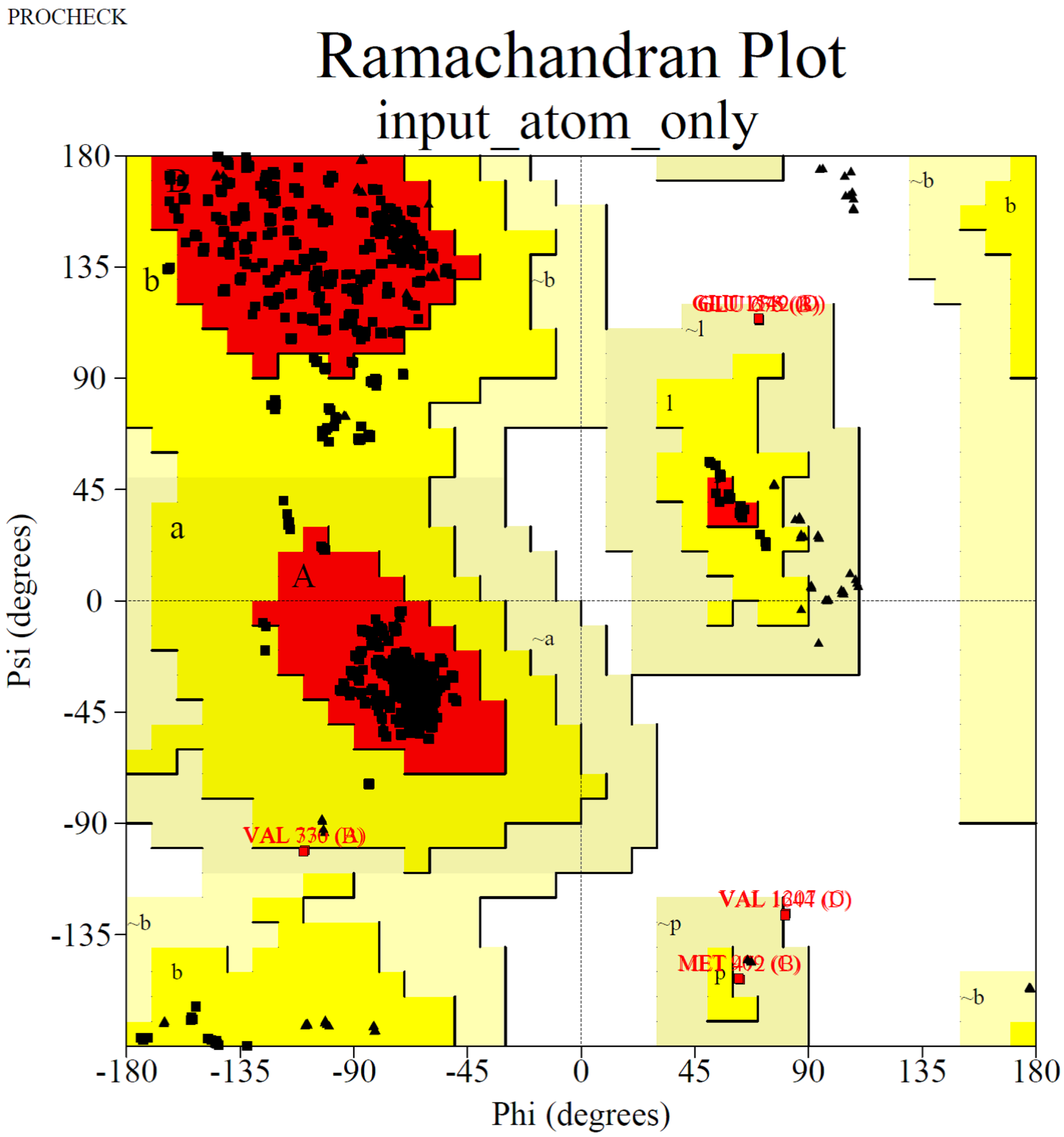

Figure S6. Ramachandran plot of *Piromyces sp.* XI theoretical model.

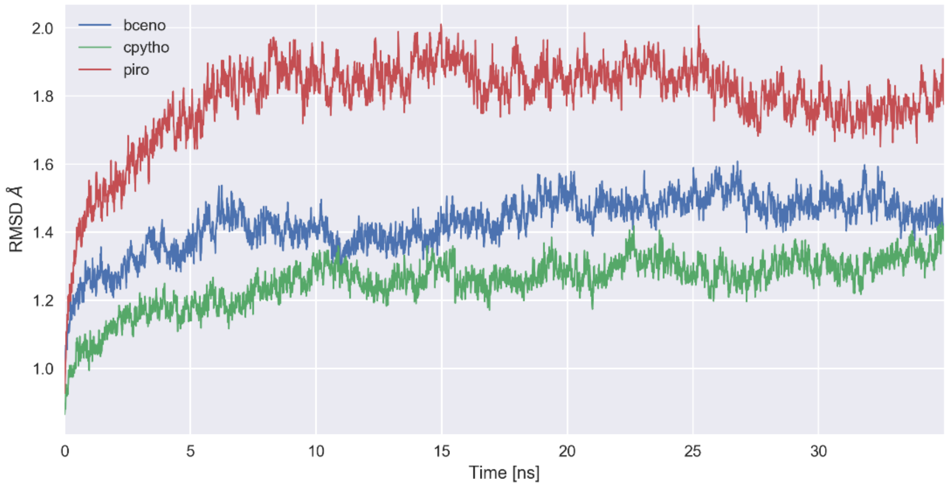

Figure S7. Global RMSD during dynamics. Blue: *B. cenocepacia*. Green: *C. phytofermentans.* Red: *Piromyces sp.*

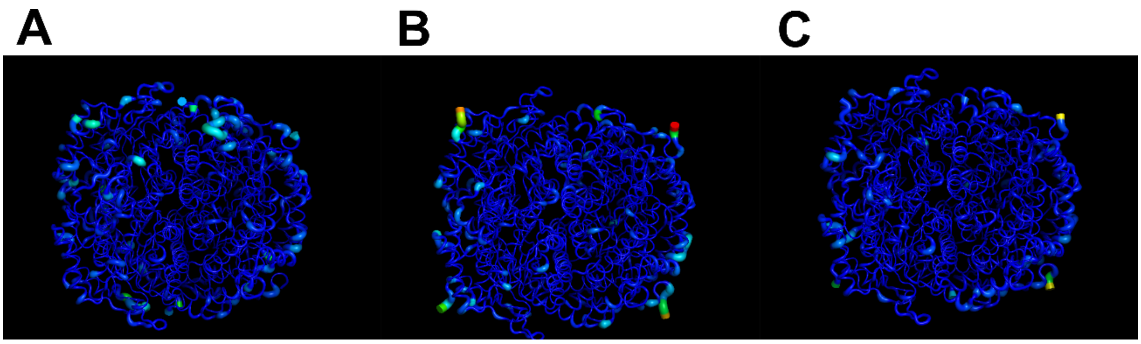

Figure S8. 3D RMS fluctuation. A) *B. cenocepacia.* B) *C. phytofermentans*. C) *Piromyces sp.* Structures are in a tube representation where thickness and color represents flexibility. Hot colors represent a higher fluctuation during dynamics simulation.

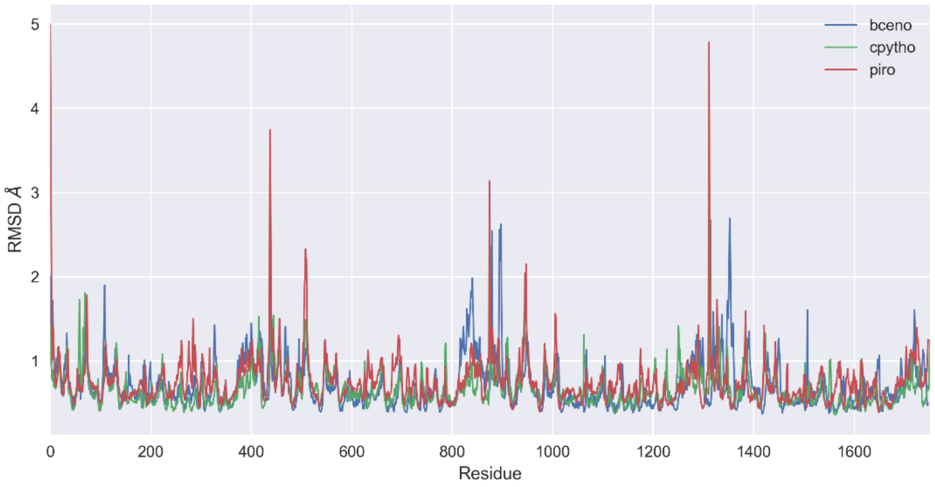

Figure S9. RMSF variation during dynamics simulation. Blue: *B. cenocepacia.* Green: *C. phytofermentans.* Red: *Piromyces sp*

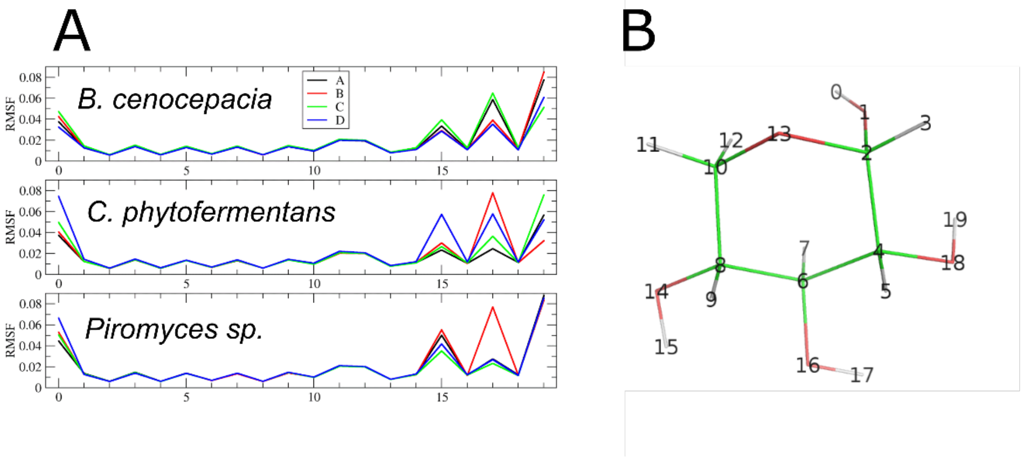

Figure S10. D-xylose RMSF inside the active site during dynamics simulation. A) RMSF for each xylose atom. B) D-xylose structure with each atom numbered.

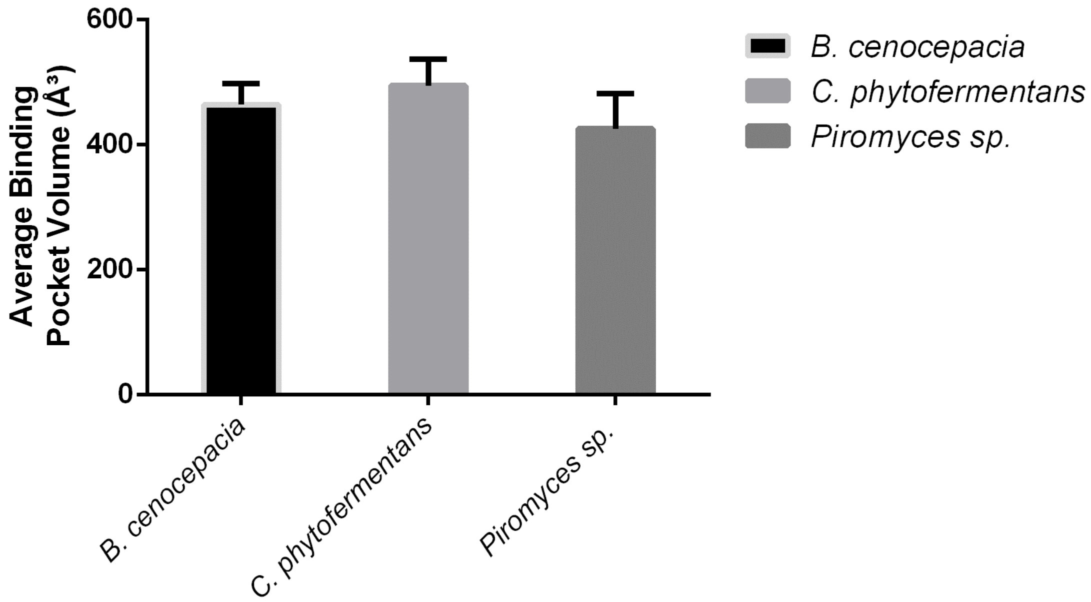

Figure S11. Binding site pocket volume. The volume of the protein binding site domain cavity was calculated from the overall average structure resulted from dynamics simulation. The column bars represent the standard error. Data shown are representative of an average of 4 domains from each protein.

```
CLUSTAL multiple sequence alignment by MUSCLE (3.8)

bcenocepacia_xyla            --MSYFEHIPAIRYEGPQSDNPLAYHHYDPDKRVLGKTLAEHLRIAVCYWHTFVWPGHDI  58
C_phytofermentans_xyla       -MKNYFPNVPEVKYEGPNSTNPFAFKYYDANKVVAGKTMKEHCRFALSWWHTLCAGGADP  59
piromyces_xyla               MAKEYFPQIQKIKFEGKDSKNPLAFHYYDAEKEVMGKKMKDWLRFAMAWWHTLCAEGADQ  60
                                .** ::  :.:** :* **:*:::**.:* * **.: :. *:*:.:***:   * *

bcenocepacia_xyla            FGQAAFRRPWQQPGDALERARMKADAAFEFFTKLGTPFYTFHDTDVAPEGDSLREYAANF  118
C_phytofermentans_xyla       FGVTTMDRTYGNITDPMELAKAKVDAGFELMTKLGIEFFCFHDADIAPEGDTFEESKKNL  119
piromyces_xyla               FGGGTKSFPWNEGTDAIEIAKQKVDAGFEIMQKLGIPYYCFHDVDLVSEGNSIEEYESNL  120
                             **  :   .: :  *.:* *. *.**.**:: ***  :: ***.*:..**::: *   *:

bcenocepacia_xyla            ARMVDYLGERQQASGVRLLWGTANLFSHPRFAAGAATNPNPDVFAWAATQVCHALDATHR  178
C_phytofermentans_xyla       FEIVDYIKEKMDQTGIKLLWGTANNFSHPRFMHGASTSCNADVFAYAAAKIKNALDATIK  179
piromyces_xyla               KAVVAYLKEKQKETGIKLLWSTANVFGHKRYMNGASTNPDFDVVARAIVQIKNAIDAGIE  180
                               :* *: *. . :*:.***.*** *.* *:  **:*. : **.* * .:: :*:**
                                                                                  ↓
bcenocepacia_xyla            LGGENYVLWGGREGYETLLNTDLKRERDQFARFLSMVVEHKHRIGFKGALLIEPKPQEPT  238
C_phytofermentans_xyla       LGGKGYVFWGGREGYETLLNTDLGLELDNMARLMKMAVEYGRANGFDGDFYIEPKPKEPT  239
piromyces_xyla               LGAENYVFWGGREGYMSLLNTDQKREKEHMATMLTMARDYARSKGFKGTFLIEPKPMEPT  240
                             **.:.**:******* :*****   * :::* ::.*. :: .  **.* : ***** ***
                                                        ↓ ↓            ↓              ↓
bcenocepacia_xyla            KHQYDYDVATVHGFLVQYGLQNEIRVNIEANHATLAGHSFHHEIANAFALGVFGSVDANR  298
C_phytofermentans_xyla       KHQYDFDTATVLAFLRKYGLEKDFKMNIEANHATLAGHTFEHELAMARVNGAFGSVDANQ  299
piromyces_xyla               KHQYDVDTETAIGFLKAHNLDKDFKVNIEVNHATLAGHTFEHELACAVDAGMLGSIDANR  300
                             ***** *. *. .**  :.*:::::.:***.********:* **:* *   * :**:***.
                                                                     ↓
bcenocepacia_xyla            GDPQNGWDTDQFPNSVEELTLAFYEILRHGGFTTGGMNFDAKVRRQSIDPEDLFYGHVGA  358
C_phytofermentans_xyla       GDPNLGWDTDQFPTDVHSATLAMLEVLKAGGFTNGGLNFDAKVRRGSFEFDDIAYGYIAG  359
piromyces_xyla               GDYQNGWDTDQFPIDQYELVQAWMEIIRGGGFVTGGTNFDAKTRRNSTDLEDIIIAHVSG  360
                             ** : ******** .  . *  *::. ***..** *****.** * : :*:  .::..

bcenocepacia_xyla            IDVLALALERAAVLVENDRLDALRRQRYAQWDDAFGRKILAGGYTLESLAADALARGVDP  418
C_phytofermentans_xyla       MDTFALGLIKAAEIIDDGRIAKFVDDRYASYKTGIGKAIVDGTTSLEELEQYVLTHS-EP  419
piromyces_xyla               MDAMARALENAAKLLQESPYTKMKKERYASFDSGIGKDFEDGKLTLEQVYEYGKKNG-EP  420
                             :*.:* .* .** ::::.    :  :***.:. .:*. :  *  :**.:       .. :*

bcenocepacia_xyla            QHASGAQERLENIVNQAIYGLR                                       440
C_phytofermentans_xyla       VMQSGRQEVLETIVNNILF--R                                       438
piromyces_xyla               KQTSGKQELYEAIV--AMY--Q                                       437
                                ** **  * **   ::  .
```

Similarity:

B. cenocepacia and *C. phytofermentans*: 52%

B. cenocepacia and *Piromyces sp*. E2: 50%

C. phytofermentans and *Piromyces sp*. E2: 54%

Figure S12. XylA protein sequence alignment. Arrows indicate substrate and metal binding sites.
